# Supplementary material for: Resolving the full spectrum of human genome variation using Linked-Reads
Source: Genome Res. 2019 Apr;29(4):635–45. doi: 10.1101/gr.234443.118 (PMC6442396; doi:10.1101/gr.234443.118)
Supplement: Supplemental Material [file supp_gr.234443.118_Supplemental_longranger-2.2.zip › longranger-2.2.tar.bz2!/lr2.2-public/lib/stan/cmdstan/stan/doc/api/html/classstan_1_1agrad_1_1chainable__alloc.html]

Stan: stan::agrad::chainable\_alloc Class Reference


|  |  |
| --- | --- |
|  | Stan  2.5.0  probability, sampling & optimization |


- Main Page
- Related Pages
- Namespaces
- Classes
- Files

- Class List
- Class Index
- Class Hierarchy
- Class Members

All Classes Namespaces Files Functions Variables Typedefs Enumerations Enumerator Friends Macros Pages

- stan
- agrad
- chainable\_alloc

Public Member Functions |
List of all members

stan::agrad::chainable\_alloc Class Reference

A chainable\_alloc is an object which is constructed and destructed normally but the memory lifespan is managed along with the arena allocator for the gradient calculation.
More...

`#include <var_stack.hpp>`

Inheritance diagram for stan::agrad::chainable\_alloc:

|  |  |
| --- | --- |
| Public Member Functions | |
|  | chainable\_alloc () |
|  | |
| virtual | ~chainable\_alloc () |
|  | |

## Detailed Description

A chainable\_alloc is an object which is constructed and destructed normally but the memory lifespan is managed along with the arena allocator for the gradient calculation.

A chainable\_alloc should never be created on the stack, only with a new call.

Definition at line 31 of file var\_stack.hpp.

## Constructor & Destructor Documentation

|  |  |  |  |  |
| --- | --- | --- | --- | --- |
| stan::agrad::chainable\_alloc::chainable\_alloc | ( |  | ) |  |

inline

Definition at line 33 of file var\_stack.hpp.

|  |  |  |  |  |
| --- | --- | --- | --- | --- |
| virtual stan::agrad::chainable\_alloc::~chainable\_alloc | ( |  | ) |  |

inlinevirtual

Definition at line 36 of file var\_stack.hpp.

---

The documentation for this class was generated from the following file:

- src/stan/agrad/rev/var\_stack.hpp


---

[ Stan Home Page ]

*© 2011–2014,
Stan Development Team.*
